# Combined Therapy with Anti-PD1 and BRAF and/or MEK Inhibitor for Advanced Melanoma: A Multicenter Cohort Study

**DOI:** 10.3390/cancers12061666

**Published:** 2020-06-23

**Authors:** Sandra Huynh, Laurent Mortier, Caroline Dutriaux, Eve Maubec, Marie Boileau, Olivier Dereure, Marie-Therese Leccia, Jean-Philippe Arnault, Florence Brunet-Possenti, Francois Aubin, Brigitte Dreno, Marie Beylot-Barry, Celeste Lebbe, Wendy Lefevre, Julie Delyon

**Affiliations:** 1 Dermatology Department, AP-HP Hôpital Saint-Louis, 75010 Paris, France; sandra.huynh@aphp.fr (S.H.); celeste.lebbe@aphp.fr (C.L.); wendy.lefevre-ext@aphp.fr (W.L.); 2 Service de Dermatologie, Universite de Lille, Inserm U1189, CHU de Lille, 59000 Lille, France; laurent.mortier@chru-lille.fr; 3 Department of Dermatology, Bordeaux Universitary Hospital, 33000 Bordeaux, France; caroline.dutriaux@chu-bordeaux.fr (C.D.); marie.beylot-barry@chu-bordeaux.fr (M.B.-B.); 4 Dermatology Department, AP-HP Hôpital Avicenne, Université Paris 13, 93000 Bobigny, France; eve.maubec@aphp.fr; 5 Service de Dermatologie, Universite de Lille, CHU de Lille, 59000 Lille, France; marie.boileau.dermato@gmail.com; 6 Department of Dermatology, University of Montpellier, 34090 Montpellier, France; o-dereure@chu-montpellier.fr; 7 Dermatology Department, Hôpital Albert Michallon, 38700 Grenoble, France; MTLeccia@chu-grenoble.fr; 8 Dermatology Department, CHU d’Amiens-Picardie Site Nord, 80080 Amiens, France; arnault.jean-philippe@chu-amiens.fr; 9 Dermatology Department, AP-HP Hôpital Bichat, 75018 Paris, France; florence.brunet-possenti@aphp.fr; 10 Dermatology Department, Hôpital Jean Minjoz, 25000 Besançon, France; francois.aubin@univ-fcomte.fr; 11 Dermatology Department, CHU de Nantes, 44000 Nantes, France; brigitte.dreno@atlanmed.fr; 12 INSERM U976, Team 1, Human Immunology Pathophysiology & Immunotherapy (HIPI), Université de Paris, F-75010 Paris, France

**Keywords:** anti-PD1, targeted therapy, BRAF inhibitor, MEK inhibitor, melanoma

## Abstract

Despite significant progress in melanoma survival, therapeutic options are still needed in case of progression under immune checkpoint inhibitors (ICI), and resistance to targeted therapies (TT) in BRAF-mutated melanomas. This study aimed to assess the safety of combined ICI and TT as a rescue line in real-life clinical practice. We conducted a study within the prospective French multicentric MelBase cohort, including patients treated with a combination of anti-PD1 (pembrolizumab/nivolumab) and BRAF inhibitor (BRAFi: dabrafenib/vemurafenib) and/or MEK inhibitors (MEKi: trametinib/cobimetinib) for BRAF mutated or wild-type advanced melanoma. Fifty-nine patients were included: 30% received the triple combination, 34% an anti-PD1 and BRAFi, and 36% an anti-PD1 and MEKi. Grade 3–4 adverse events occurred in 12% of patients. Permanent discontinuation or dose reduction of one of the treatments for toxicity was reported in 14% and 7% of patients, respectively. In the BRAF wild-type subgroup, treatment with MEKi and anti-PD1 induced a tumor control rate of 83% and median progression-free survival of 7.1 months. The combination of anti-PD1 and BRAFi and/or MEKi was a safe rescue line for advanced melanoma patients previously treated with ICI/TT. The benefit of these combinations, specifically anti-PD1 and MEKi in BRAF wild-type melanoma patients, needs to be prospectively studied.

## 1. Introduction

Tyrosine kinase inhibitors (TKI) targeting mutated BRAF and immune checkpoint inhibitors (ICI) have revolutionized advanced melanoma treatment and prognosis. However, clinicians still have to face significant issues, such as fast initial response to TKI but the acquisition of resistance mechanisms with frequent secondary progression, and a lower response rate, and delayed tumoral response to anti-programmed cell death 1 (anti-PD1) or anti-cytotoxic T-lymphocyte associated protein 4 (anti-CTLA-4).

In BRAF-mutated melanoma, the response rate to BRAF and MEK inhibition was 67–69% [1,2,3] and 68–70% [4,5] with first-line dabrafenib + trametinib and vemurafenib + cobimetinib, respectively; and 63% with encorafenib + binimetinib in previously untreated patients or who progressed after previous ICI [6]. However, the progression-free survival (PFS) ranged from 11 to 14.9 months [2,5,6], highlighting the median time to the development of acquired resistance. In NRAS-mutated melanomas, ICI are the only therapeutic option that proved efficacy in overall survival (OS). Treatment with a MEK inhibitor (MEKi) was disappointing, where phase 3 NEMO study showed a modest increase of PFS with binimetinib (2.8 months) versus dacarbazine (1.7 months) in NRAS-mutated advanced melanoma [7] such that MEKi is rarely used as a valuable option in this setting. 

With ICI, the response rate to first or second-line anti-PD1 in BRAF-mutated or wild-type (WT) melanomas ranged from 42% [8] to 45% [9,10,11], with a median PFS of 8.4 to 9.7 [8] and 6.9 months [9,10,11] with pembrolizumab and nivolumab, respectively. A higher response rate of 58% was reported with first-line combined ipilimumab + nivolumab, but grade 3 and 4 treatment-related adverse events (AEs) occurred in more than half of the patients (59%) [11]. 

In case of progression with both treatment classes, if the inclusion in a clinical trial is not possible, the combination of ICI with BRAF inhibitor (BRAFi) and/or MEKi may be an option, especially if progression is limited to 1 or 2 metastatic sites and is sometimes used in clinical practice. The rationale of ICI and BRAFi and/or MEKi combinations was first studied in vitro and in vivo. It relies on a better tumor recognition by lymphocytes after BRAF or combined BRAF and MEK inhibition through several mechanisms such as tumor antigens release following tumor cell death, increased expression of melanocyte differentiation antigens, increased tumor T-cell infiltration and cytotoxic activity, and decreased immunosuppressive cytokines in the tumor’s microenvironment [12,13,14,15,16,17,18,19,20,21]. Moreover, adding MEKi to ICI could limit immune-related resistance mechanisms, i.e., BRAFi-resistant melanoma cells had increased PD-L1 expression that could be reversed by MEKi addition [13,17]. A combination of ICI with BRAFi and MEKi in BRAF-mutated melanomas was then developed in phase 1 studies. While the combination with ipilimumab was too toxic [22,23], phase III clinical trials comparing the triple combination of anti-PD1 with dabrafenib + trametinib versus dabrafenib + trametinib + placebo (Keynote-022 [24], combi-I [25]), or the anti-programmed cell death ligand 1 (anti-PDL1) atezolizumab with vemurafenib + cobimetinib versus vemurafenib + cobimetinib + placebo (TRILOGY IMspire150 [26]) in treatment-naïve patients with BRAF V600-mutated advanced melanoma are ongoing. In BRAF WT melanoma, few data have been published to date about the combination of MEKi with anti-PD1 [27]. 

The combination of BRAF and/or MEK inhibitors with anti-PD1 is a therapeutic alternative sometimes used in clinical practice in BRAF mutated or WT melanoma when no other approved therapeutic option or clinical trial is available. We conducted a multicenter study to assess the safety of these combinations in real-life clinical practice.

## 2. Results

### 2.1. Patients’ Characteristics and Treatment

Between 27 October 2014 and 1 January 2019, 61 patients from 10 French centers received the combination of an anti-PD1 with a BRAFi and/or a MEKi for advanced melanoma outside of a clinical trial. Two patients were excluded from analysis because of missing data; and this study finally included 59 patients. 

Patients’ characteristics at baseline are detailed in Table 1. There were 32 men (54%) and 27 women (46%), with a median age of 57 years old (27–88). All patients had stage IV melanoma according to the AJCC 7th edition and had previously received at least one systemic treatment. Six patients (10%) had an ECOG > 1, 13 (22%) had elevated LDH, and 29 (49%) had 3 or more metastatic sites, including 30 patients (51%) with brain metastasis. Nineteen patients (32%) received the combination of an anti-PD1 and BRAFi and/or MEKi as their last therapeutic line. The main reason for starting combined therapy was progression in 76% of patients.

Eighteen patients (30%) received a triple-combination of anti-PD1 + BRAFi + MEKi, 20 patients (34%) an anti-PD1 + BRAFi (all BRAF-mutated), and 21 (36%) an anti-PD1 + MEKi (Table 2 and Table S1).

Forty patients (68%) had BRAF V600-mutated melanoma. Among them, 18 received a triple-combination, 20 received a combination of anti-PD1 + BRAFi, and two had a combination of anti-PD1 + MEKi (Table 2). Thirty-eight patients (95%) were previously treated with TKI monotherapy or BRAFi and MEKi combination, 16 (40%) with anti-PD1, eight (20%) with combined ipilimumab and nivolumab, and 2 (5%) with ipilimumab.

Eighteen patients (30%) had BRAF WT melanoma, among whom 13 were NRAS-mutated. All of them received an anti-PD1 + MEKi combination (Table 2). Fourteen patients (78%) had previously been treated with anti-PD1, seven (39%) with ipilimumab, and five (28%) with combined ipilimumab and nivolumab. Two of them (11%) had received a single MEKi before the combination.

### 2.2. Safety

The median follow-up duration was 5 months (0–45). The median treatment duration with the combination was 2 months (0–48). The median time from starting combination to any AE occurrence was 34 days (1–436): 24 days (3–127) with the triple-combination, versus 53 days (5–436) with anti-PD1 + BRAFi combination, and 40 days (1–373) with the anti-PD1 + MEKi combination.

At least one any-grade treatment-related AE occurred in 33 patients (56%): 11 (61%) with the triple-combination, 9 (45%) with-PD1 + BRAFi, and 13 (62%) with anti-PD1 and MEKi. Grade 3/4 AEs occurred in seven patients (12%), i.e., four with the triple- combination and three with anti-PD1 + MEKi.

The main treatment-related AEs are listed in Table 3 (refer to Supplementary Table S2 for all treatment-related AEs). Skin toxicity, fever, and diarrhea were the most frequent AEs. Grade 3/4 AEs were aspartate aminotransferase (AST) or creatine phosphokinase elevation, hyponatremia, diarrhea, muscle weakness, or skin toxicity (acneiform or maculopapular rash). Ten percent of patients were hospitalized for treatment toxicity, and there was no treatment-related death. Fever was more frequent with the triple-combination compared to double-combinations (39% versus 15% and 0% with the double combinations). Patients treated with an anti-PD1 + MEKi experienced more cutaneous (43%, versus 25% with anti-PD1 + BRAFi and 17% with the triple combination), digestive (29%, versus 10% and 11%, respectively) and respiratory (19% versus 0% and 0%, respectively) AEs.

At least one immune-related adverse event (irAE), i.e., due to either nivolumab or pembrolizumab, was recorded in 14 patients (24%). The most frequent reported irAEs were fever in eight patients (13%), diarrhea in four patients (7%), followed by chills, hypothyroidism, pneumonitis, pruritus (3% each). 

Permanent interruption of a study drug because of toxicity occurred in eight patients (14%), where five of them received the triple-combination, and three an anti-PD1 + MEKi. Temporary discontinuation of one of the treatments for toxicity was reported in 6 patients (10%). Four patients (7%) required dose reduction of at least one treatment. Only one patient, treated with the triple-combination, required systemic corticosteroid.

### 2.3. Efficacy

#### 2.3.1. Efficacy in BRAF-Mutated Melanoma Patients

**Figure 1:**
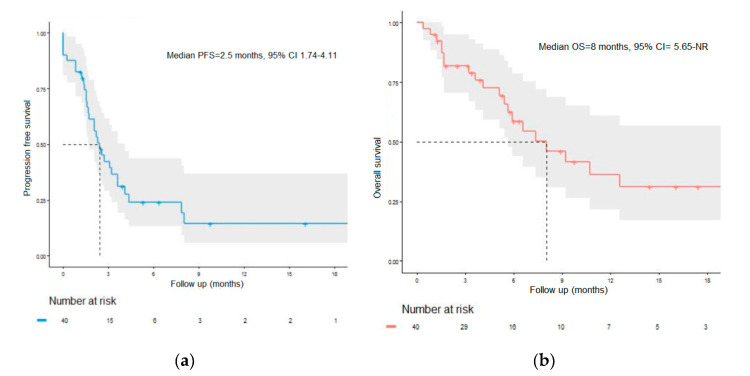
Survival in the BRAF-mutant subgroup. (**a**) Progression-free survival in the BRAF-mutant subgroup. PFS: progression-free survival. (**b**) Overall survival in the BRAF-mutant subgroup. OS: overall survival; NR: not reached.

The objective response rate was 12%, and the disease control rate was 52% in the BRAF-mutated subgroup (Table 4). The median PFS was 2.5 months (95% CI = 1.74–4.11), with a 12-month PFS rate of 14.9% (95% CI = 5.9–37.3) (Figure 1a). The median OS was 8 months (95% CI = 5.7–not reached), with a 12-month OS rate of 36% (95% CI = 21.6–61.1) (Figure 1b)

#### 2.3.2. Efficacy in BRAF-WT Melanoma Patients

**Figure 2:**
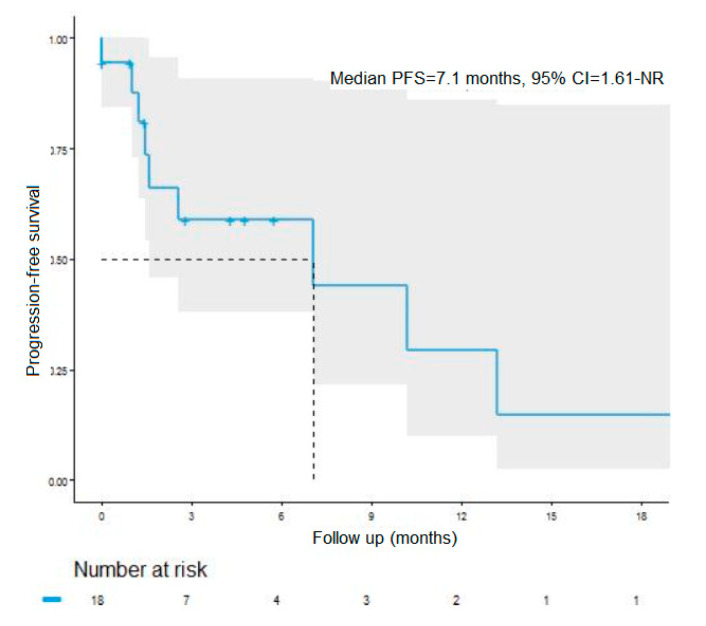
Progression-free survival in the BRAF-wild type subgroup. PFS: progression-free survival; NR: not reached.

The objective response rate was 11%, and the disease control rate was 83% in the BRAF WT subgroup (Table 4). The median PFS was 7.1 months (95 CI% = 1.6-not reached), with a 12-month PFS rate of 27.5% (95% CI = 9.3–81.0) (Figure 2). The median OS was 10.2 months (95% CI = 5.5–not reached), with a 12-month OS rate of 35% (95% CI = 12.1–100) (data not shown due to a very small number of events in this subgroup).

## 3. Discussion

This real-life clinical practice study aimed to evaluate the safety of a “rescue-line” with combined anti-PD1 and BRAFi and/or MEKi after failure or limiting the toxicity of first-line treatments (TKI and/or ICI). Severe (grade 3 or 4) AEs occurred in only 12% of the patients, with a disease control rate of 52% in the BRAF-mutated patients receiving 3 possible combinations (anti-PD1 + BRAFi + MEKi or anti-PD1 + BRAFi or anti-PD1 + MEKi), and 83% in the BRAF-WT patients receiving anti-PD1 + MEKi. The combined therapy was initiated in patients with aggressive advanced melanoma (3 or more metastatic sites, ECOG > 1, or high LDH level), half of them harboring brain metastasis, all previously treated with TKI (for BRAF-mutated melanoma patients) or ICI. This strategy was chosen either because of progressive disease or the occurrence of limiting grade 3/4 AEs with previous lines, in patients not eligible for clinical trials. The combination therapy was the last therapeutic option in a third of the patients.

The primary endpoint of the safety assessment revealed an acceptable toxicity profile. Fever was more frequent with the triple combination compared to double-combinations, and cutaneous, digestive, and respiratory AEs occurred more frequently in patients treated with anti-PD1 + MEKi. Interestingly, the rate of grade 3 or 4 AEs was lower in our study than in the phase 2 study Keynote-022 [24], a randomized, double-blind trial comparing the triple-combination of dabrafenib + trametinib + pembrolizumab versus dabrafenib + trametinib + placebo as first-line treatment. Severe (grade 3 or more) treatment-related AEs rate was 58% in the triplet arm, mostly fever, increased transaminase, and rash vs. 7% in the present series. One patient receiving the triplet died of pneumonitis. Similarly, dose reductions and temporary or permanent treatment discontinuations were less frequent in our series than in Keynote-022. In other prospective clinical trials assessing the triple combination, grade 3/4 AEs occurred respectively in 75% (spartalizumab + dabrafenib + trametinib as first-line in COMBI-I trial [25]), 67% (atezolizumab + vemurafenib + cobimetinib as first-line in TRILOGY IMspire 150 trial [26]), and 65% of patients (nivolumab + dabrafenib + trametinib, as first-line TKI in TRIDeNT trial [28]). Differences between these studies and ours regarding safety could be explained because most patients in our study had been previously treated with anti-PD1 or TKI and were considered eligible for reintroduction. Conversely, patients who had experienced prior severe toxicity with one of those treatments were probably not subsequently treated with any combination of anti-PD1 +- TKI. Moreover, drugs were introduced sequentially in our study as most of the patients had previously received anti-PD1 and/or BRAFi or MEKi, whereas they were associated upfront as first-line treatment in Keynote-022, COMBI-I and TRILOGY IMspire 150 [24,25,26].

In the BRAF WT subgroup of our study, including 72% of patients harboring an NRAS-mutation, the median PFS was 7.1 months with combined anti-PD1 and MEKi, although all patients had previously been treated with an ICI and 28% with ipilimumab + nivolumab. Comparatively, in the NEMO study, the median PFS was 2.8 months with binimetinib for patients with NRAS-mutated melanomas who were previously untreated or had progressed after previous immunotherapy [7]. Interestingly, the median PFS with binimetinib was longer in patients previously treated with immunotherapy compared to those who did not (5.5 versus 2.8 months, respectively). Similarly, Kirchberger et al. reported a trend toward an increase of OS in patients with NRAS-mutated melanoma treated with ICI who received a MEKi before, during, or after ICI, compared to MEKi-naïve patients (25 versus 20 months respectively) [29]. These data, combined with our results, support a rationale for combining immunotherapy and MEKi in NRAS-mutated melanoma. However, the results of the IMspire 170 phase III trial comparing atezolizumab + cobimetinib to pembrolizumab in 450 patients with untreated BRAF WT melanoma proved negative [27]. The group of patients with BRAF WT and NRAS WT was too limited in size to be individually analyzed. Additional studies are warranted to define the place of MEKi and ICI in BRAF-WT melanoma.

In the BRAF-mutated subgroup, the response rate was low at 12%, and the disease control rate was 52%. All the patients had previously received one or more therapeutic lines, including BRAFi and MEKi for most of them, and the combination therapy with anti-PD1 and TKI targeting mutated BRAF being the last therapeutic option in 35% of the BRAF-mutated patients. Comparatively, Keynote-022 showed a response rate of 63% in the triplet arm in previously untreated patients. The median PFS was numerically superior in the triplet arm (16 months) compared to the doublet arm (10 months), but the difference did not reach statistical significance [24]. TRIDeNT study showed a 91% response rate, where 100% of PD1-naïve patients responded versus 83% of PD1-refractory patients [28]. We may hypothesize that acquired resistance to previous treatment with BRAFi and MEKi could not be overcome by the addition of an anti PD1 in our study, in which patients had severely advanced disease.

The retrospective study design limits the interpretation of our results, the lack of a control arm, as well as the small size of patients’ subsets with a heterogeneous cohort, including BRAF-mutated and BRAF WT-melanomas treated with different combination therapies. The low rate of patients previously treated with ipilimumab + nivolumab, which recently became the standard first-line, needs to be taken into consideration in the interpretation of the results. However, our study showed that combining anti-PD1 with BRAFi and/or MEKi might be an interesting rescue line, especially in patients with advanced BRAF WT melanomas for whom therapeutic options are limited, with tumor control achieved in more than half of patients and relatively good tolerance. Still, the questions of the optimal combination, and the interest of sequenced-therapy with a run-in period with BRAFi and/or MEKi before ICI remain to be determined in prospective studies.

## 4. Materials and Methods

### 4.1. Study Design

We conducted an observational study within MelBase, a nationwide French clinical database dedicated to the prospective follow-up of adult patients with advanced melanoma (unresectable stage III or advanced stage IV, according to the American Joint Committee on Cancer (AJCC) 7th edition), naïve of non-adjuvant systemic treatment, and active since 2013 in 26 participating French centers. The French ethics committee approved MelBase protocol (Comité de protection des personnes Ile-de-france XI, *n*°12,027, 2012) and registered in the National Institute of Health clinical trials database (NCT02828202). Written informed consent was obtained from all patients.

### 4.2. Patients

Inclusion criteria were MelBase patients treated with a combination of an anti-PD1 (nivolumab or pembrolizumab) with a BRAFi (dabrafenib, vemurafenib) and/or a MEKi (trametinib, cobimetinib) according to investigator’s choice, until 1 January 2019, outside of a clinical trial.

### 4.3. Procedure

For each patient, following data were retrospectively recorded: demographics (gender, age at studied therapeutic combination initiation), previous therapeutic lines, melanoma genotype (BRAF V600 or WT, NRAS mutated or WT), melanoma characteristics at combined therapy initiation (time from advanced/metastatic melanoma diagnosis to the beginning of combined therapy, AJCC staging 7th edition, metastatic sites (<3, or ≥3), brain metastasis, Eastern Cooperative Oncology Group Performance Status (ECOG, ≤1 or >1), Lactic-DeHydrogenase (LDH) level), nature of the therapeutic combination received, duration of treatment with combined therapy and duration of follow up. Data were extracted on 21 February 2019.

### 4.4. Outcomes and Endpoints

The primary endpoint was the safety assessment of combined anti-PD1 and BRAF and/or MEK inhibitors. The main criterion was the occurrence of severe, grade 3, or more, AEs, according to the National Cancer Institute Common Terminology Criteria for Adverse Events, version 4.1. Secondary criteria were the any-grade AEs rate, temporary/permanent discontinuation proportion, dose reduction proportion, the proportion of patients requiring systemic corticosteroid, and main AEs classes. Only treatment-related AEs were considered.

The secondary endpoint was the efficacy assessment in 2 distinct cohorts, i.e., patients with BRAF-mutated or BRAF WT melanoma. The center investigator assessed the tumoral response according to RECIST (Response Evaluation Criteria in Solid Tumors) version 1.1. OS and PFS were estimated using a Kaplan–Meier analysis.

## 5. Conclusions

Combined therapy with anti-PD1 and BRAFi and/or MEKi is a rescue line with acceptable tolerance in patients with advanced melanoma previously treated with BRAFi and/or MEKi or ICI. The combination of anti-PD1 and MEKi in the BRAF WT subgroup, previously treated with ICI, showed an interesting disease control rate supporting the need for further studies with larger samples.

## Figures and Tables

**Table 1:** Patients’ baseline characteristics at combined anti-PD1 + BRAF inhibitor and/or MEK inhibitor initiation.

Characteristics	Total *n* (%) (*n* = 59)	BRAF-Mutated, *n* (%) (*n* = 40)	BRAF-Wildtype, *n* (%) (*n* = 18)
Median age, years (range)	57 (27–88)	54 (27–78)	72 (37–88)
Gender:			
Male	32 (54)	20 (50)	12 (67)
Female	27 (46)	20 (50)	6 (33)
ECOG ^1^			
≤1	51 (86)	35 (87)	15 (83)
>1	6 (10)	4 (10)	2 (11)
NS ^2^	2 (3)	1 (2)	1 (6)
AJCC staging ^3^			
IV M1a	2 (3)	0 (0)	2 (11)
IV M1b	7 (12)	3 (8)	3 (17)
IV M1c	50 (85)	37 (92)	13 (72)
Number of metastatic sites			
<3	25 (42)	18 (45)	6 (33)
≥3	29 (49)	18 (45)	11 (61)
NS ^2^	5 (8)	4 (10)	1 (6)
Brain metastasis	30 (51)	22 (55)	8 (44)
LDH ^4^			
Normal	37 (63)	24 (60)	12 (67)
High	13 (22)	9 (22)	4 (22)
NS ^2^	9 (15)	7 (18)	2 (11)
Median time from advanced melanoma diagnosis to onset of combination therapy, months (range)	12 (1–127)	10 (1–61)	13 (3–57)
Reason for combination therapy initiation			
Progression	45 (76)	29 (72.5)	16 (89)
Toxicity	3 (5)	3 (7.5)	0
NS ^2^	11 (19)	8 (20)	2 (11)
Previous lines			
Ipilimumab + nivolumab	13 (22)	8 (20)	5 (28)
Ipilimumab	9 (15)	2 (5)	7 (39)
Anti-PD1	30 (51)	16 (40)	14 (78)
BRAFi ^5^ + MEKi ^6^	33 (56)	33 (83)	0
BRAFi ^5^	11 (19)	11 (28)	0
MEKi ^6^	3 (5)	1 (3)	2 (11)
Chemotherapy	7 (12)	1 (3)	6 (33)

^1^ ECOG: Eastern Cooperative Oncology Group; ^2^ NS: not specified; ^3^ AJCC: American Joint Committee on Cancer 7th edition; ^4^ LDH: lactate dehydrogenase; ^5^ BRAFi: BRAF inhibitor; ^6^ MEKi: MEK inhibitor. Comment: total *n* can be different from BRAF-mutated *n* + BRAF-wildtype *n* because one patient had equivocal BRAF mutational status.

**Table 2:** Type of drug combination depending on the BRAF mutational status.

BRAF Status	Anti-PD1 + BRAFi ^1^ + MEKi ^2^, *n*	Anti-PD1 + BRAFi ^1^, *n*	Anti-PD1 + MEKi ^2^, *n*	Total, *n*
BRAF-mutant	18	20	2	40
BRAF-wildtype	0	0	18	18
Unknown	0	0	1	1
Total, *n*	18	20	21	59

^1^ BRAFi: BRAF inhibitor; ^2^ MEKi: MEK inhibitor.

**Table 3:** Main treatment-related adverse events.

Event	Anti-PD1 + BRAFi ^1^ + MEKi ^2^ (*n* = 18)		Anti-PD1 + BRAFi ^1^ (*n* = 20)		Anti-PD1 + MEKi ^2^ (*n* = 21)	
	Any Grade	Grade 3–4	Any Grade	Grade 3–4	Any Grade	Grade 3–4
	Number of Patients with Event (%)					
**AE ^3^ occurring in ≥10% of total patients**						
Pyrexia	7 (39)	0 (0)	3 (15)	0 (0)	0 (0)	0 (0)
Diarrhea	1 (6)	1 (6)	1 (5)	0 (0)	5 (24)	0 (0)
Acneiform rash	0 (0)	0 (0)	1 (5)	0 (0)	6 (29)	1 (5)
AE ^3^ of interest occurring in <10% of total patients *						
Skin and subcutaneous tissue disorders—other **	2 (11)	1 (6)	2 (10)	0 (0)	1 (5)	0 (0)
Fatigue	2 (11)	0 (0)	1 (5)	0 (0)	1 (5)	0 (0)
Chills	3 (17)	0 (0)	1 (5)	0 (0)	0 (0)	0 (0)
Hypothyroidism	1 (6)	0 (0)	1 (5)	0 (0)	1 (5)	0 (0)
Increased CPK ^4^	0 (0)	0 (0)	0 (0)	0 (0)	3 (14)	1 (5)
Dry skin	1 (6)	0 (0)	1 (5)	0 (0)	1 (5)	0 (0)
Eosinophilia	0 (0)	0 (0)	1 (5)	0 (0)	2 (10)	0 (0)
Myalgia	1 (6)	0 (0)	0 (0)	0 (0)	2 (10)	0 (0)
Increased AST ^5^	2 (11)	1 (6)	0 (0)	0 (0)	0 (0)	0 (0)
Generalized muscle weakness	0 (0)	0 (0)	0 (0)	0 (0)	1 (5)	1 (5)
Hyponatremia	1 (6)	1 (6)	0 (0)	0 (0)	0 (0)	0 (0)
Maculopapular rash	1 (6)	1 (6)	0 (0)	0 (0)	0 (0)	0 (0)

*n* (%) represents the number of patients with an event. * among AEs occurring in less than 10% of patients: only the grade 3 or 4 AEs, and the AEs occurring in 5 to 10% of total patients are presented. Refer to Supplementary Table S2 for all treatment-related AEs. ** cheilitis (grade 3–4), folliculitis, seborrheic keratosis, palmoplantar keratoderma, pruriginous rash.^1^ BRAFi: BRAF inhibitor; ^2^ MEKi: MEK inhibitor; ^3^ AE: adverse events; ^4^ CPK: creatine phosphokinase; ^5^ AST: aspartate aminotransferase.

**Table 4:** Tumor response in BRAF-mutated or BRAF-wild type subgroups.

Tumor Response	Total (*n* = 59)	BRAF-Mutated Melanoma Patients (*n* = 40)	BRAF-Wild-Type Melanoma Patients (*n* = 18)
Best overall response, *n* (%)			
CR ^1^	2 (3)	2 (5)	0 (0)
PR ^2^	5 (8)	3 (8)	2 (11)
SD ^3^	30 (51)	16 (40)	13 (72)
PD ^4^	22 (37)	19 (48)	3 (17)
Objective overall response, *n* (%)			
CR ^1^ + PR ^2^	7 (12)	5 (12)	2 (11)
Disease control, *n* (%)			
CR ^1^ + PR ^2^ + SD ^3^	37 (63)	21 (52)	15 (83)

^1^ CR: complete response; ^2^ PR: partial response; ^3^ SD: stable disease; ^4^ PD: progressive disease.

## Supplementary Materials

The following are available online at https://www.mdpi.com/2072-6694/12/6/1666/s1, Table S1: Details of drugs received, Table S2: Exhaustive list of treatment-related adverse events.
