# Supplementary material for: Combined Therapy with Anti-PD1 and BRAF and/or MEK Inhibitor for Advanced Melanoma: A Multicenter Cohort Study
Source: Cancers (Basel). 2020 Jun 23;12(6):1666. doi: 10.3390/cancers12061666 (PMC7352575; doi:10.3390/cancers12061666)
Supplement: Supplementary file 1 [file cancers-12-01666-s001.pdf]

Supplementary Materials: Combined Therapy with Anti-PD1 and BRAF and/or MEK Inhibitor for Advanced Melanoma: A Multicenter Cohort Study

Sandra Huynh, Laurent Mortier, Caroline Dutriaux, Eve Maubec, Marie Boileau, Olivier Dereure, Marie-Therese Leccia, Jean-Philippe Arnault, Florence Brunet-Possenti, Francois Aubin, Brigitte Dreno, Marie Beylot-Barry, Celeste Lebbe, Wendy Lefevre and Julie Delyon

Table S1. Detail of drugs received.

Drug	Total *n* (%) (*n* = 59)	Anti-PD1 + BRAFi[1] + MEKi[2], *n* (%) (*n* = 18)	Anti-PD1 + BRAFi[1], *n* (%) (*n* = 20)	Anti-PD1 + MEKi[2], *n* (%) (*n* = 21)
dabrafenib	34 (58)	16 (89)	18 (90)	0 (0)
vemurafenib	4 (7)	2 (11)	2 (10)	0 (0)
trametinib	33 (56)	16 (89)	0 (0)	17 (81)
cobimetinib	6 (10)	2 (11)	0 (0)	4 (19)
nivolumab	11 (19)	3 (17)	2 (10)	6 (29)
pembrolizumab	48 (81)	15 (83)	18 (90)	15 (71)

[1]BRAFi: BRAF inhibitor; [2]MEKi: MEK inhibitor.

Table S2. Exhaustive list of treatment-related adverse events.

Event	Total Population (*n* = 59)	Anti-PD1 + BRAFi[1] + MEKi[2] (*n* = 18)		Anti-PD1 + BRAFi[1] (*n* = 20)		Anti-PD1 + MEKi[2] (*n* = 21)	
	Any grade	Any grade	Grade 3–4	Any grade	Grade 3–4	Any grade	Grade 3–4
	Number of patients with event (%)						
Acneiform rash	7 (12)	0 (0)	0 (0)	1 (5)	0 (0)	6 (29)	1 (5)
Other skin and subcutaneous tissue disorders*	5 (8)	2 (11)	1 (6)	2 (10)	0 (0)	1 (5)	0 (0)
Dry skin	3 (5)	1 (6)	0 (0)	1 (5)	0 (0)	1 (5)	0 (0)
Pruritus	2 (3)	0 (0)	0 (0)	1 (5)	0 (0)	1 (5)	0 (0)
Skin infection	2 (3)	0 (0)	0 (0)	0 (0)	0 (0)	2 (10)	0 (0)
Palmoplantar erythrodysesthesia	2 (3)	0 (0)	0 (0)	0 (0)	0 (0)	2 (10)	0 (0)
Maculopapular rash	1 (2)	1 (6)	0 (0)	0 (0)	0 (0)	0 (0)	0 (0)
Pustular rash	1 (2)	0 (0)	0 (0)	0 (0)	0 (0)	1 (5)	0 (0)
Bullous dermatitis	1 (2)	0 (0)	0 (0)	1 (5)	0 (0)	0 (0)	0 (0)
Squamous cell carcinoma	1 (2)	0 (0)	0 (0)	1 (5)	0 (0)	0 (0)	0 (0)
Skin hypopigmentation	1 (2)	0 (0)	0 (0)	1 (5)	0 (0)	0 (0)	0 (0)
Skin ulceration	1 (2)	0 (0)	0 (0)	0 (0)	0 (0)	1 (5)	0 (0)
Localized edema	1 (2)	0 (0)	0 (0)	0 (0)	0 (0)	1 (5)	0 (0)
Pyrexia	10 (17)	7 (39)	0 (0)	3 (15)	0 (0)	0 (0)	0 (0)
Chills	4 (7)	3 (17)	0 (0)	1 (5)	0 (0)	0 (0)	0 (0)
Fatigue	4 (7)	2 (11)	0 (0)	1 (5)	0 (0)	1 (5)	0 (0)
Flu like symptoms	1 (2)	1 (6)	0 (0)	0 (0)	0 (0)	0 (0)	0 (0)
Oral pain	1 (2)	0 (0)	0 (0)	0 (0)	0 (0)	1 (5)	0 (0)
Pain	1 (2)	0 (0)	0 (0)	1 (5)	0 (0)	0 (0)	0 (0)
Confusion	1 (2)	1 (6)	0 (0)	0 (0)	0 (0)	0 (0)	0 (0)
Anorexia	1 (2)	0 (0)	0 (0)	1 (5)	0 (0)	0 (0)	0 (0)
Granulomatosis	1 (2)	1 (6)	0 (0)	0 (0)	0 (0)	0 (0)	0 (0)
Diarrhea	7 (12)	1 (6)	1 (6)	1 (5)	0 (0)	5 (24)	0 (0)
Increased AST[3]	2 (3)	2 (11)	1 (6)	0 (0)	0 (0)	0 (0)	0 (0)

Increased ALT[4]	2 (3)	2 (11)	0 (0)	0 (0)	0 (0)	0 (0)	0 (0)
Increased alkaline phosphatase	2 (3)	1 (6)	0 (0)	1 (5)	0 (0)	0 (0)	0 (0)
Increased GGT[5]	2 (3)	2 (11)	0 (0)	0 (0)	0 (0)	0 (0)	0 (0)
Nausea	2 (3)	1 (6)	0 (0)	1 (5)	0 (0)	0 (0)	0 (0)
Vomiting	1 (2)	1 (6)	0 (0)	0 (0)	0 (0)	0 (0)	0 (0)
Colitis	1 (2)	1 (6)	0 (0)	0 (0)	0 (0)	0 (0)	0 (0)
Myalgia	3 (5)	1 (6)	0 (0)	0 (0)	0 (0)	2 (10)	0 (0)
Increased CPK[6]	3 (5)	0 (0)	0 (0)	0 (0)	0 (0)	3 (14)	1 (5)
Myositis	1 (2)	0 (0)	0 (0)	0 (0)	0 (0)	1 (5)	0 (0)
Rhabdomyolysis	1 (2)	0 (0)	0 (0)	0 (0)	0 (0)	1 (5)	0 (0)
Generalized muscle weakness	1 (2)	0 (0)	0 (0)	0 (0)	0 (0)	1 (5)	1 (5)
Arthralgia	1 (2)	0 (0)	0 (0)	1 (5)	0 (0)	0 (0)	0 (0)
Pneumonitis	2 (3)	0 (0)	0 (0)	0 (0)	0 (0)	2 (10)	0 (0)
Dyspnea	2 (3)	0 (0)	0 (0)	0 (0)	0 (0)	2 (10)	0 (0)
Pulmonary opacities	1 (2)	0 (0)	0 (0)	0 (0)	0 (0)	1 (5)	0 (0)
Sinus tachycardia	1 (2)	0 (0)	0 (0)	0 (0)	0 (0)	1 (5)	0 (0)
Headache	2 (3)	1 (6)	0 (0)	1 (5)	0 (0)	0 (0)	0 (0)
Meningismus	1 (2)	1 (6)	0 (0)	0 (0)	0 (0)	0 (0)	0 (0)
Tinnitus	1 (2)	0 (0)	0 (0)	1 (5)	0 (0)	0 (0)	0 (0)
Vertigo	1 (2)	1 (6)	0 (0)	0 (0)	0 (0)	0 (0)	0 (0)
Red eye	1 (2)	0 (0)	0 (0)	1 (5)	0 (0)	0 (0)	0 (0)
Hypothyroidism	3 (5)	1 (6)	0 (0)	1 (5)	0 (0)	1 (5)	0 (0)
Hyperthyroidism	1 (2)	0 (0)	0 (0)	1 (5)	0 (0)	0 (0)	0 (0)
Eosinophilia	3 (5)	0 (0)	0 (0)	1 (5)	0 (0)	2 (10)	0 (0)
Increased creatinine	2 (3)	1 (6)	0 (0)	1 (5)	0 (0)	0 (0)	0 (0)
Hyponatremia	1 (2)	1 (6)	1 (6)	0 (0)	0 (0)	0 (0)	0 (0)
Hypokalemia	1 (2)	0 (0)	0 (0)	0 (0)	0 (0)	1 (5)	0 (0)
Decreased neutrophil count	1 (2)	1 (6)	0 (0)	0 (0)	0 (0)	0 (0)	0 (0)

[1]BRAFi: BRAF inhibitor; [2]MEKi: MEK inhibitor; [3]AST: aspartate aminotransferase; [4]ALT: alanine aminotransferase; [5]GGT: gamma-glutamyltransferase; [6]CPK: creatine phosphokinase. *cheilitis (grade 3-4), folliculitis, seborrheic keratosis, palmoplantar keratoderma, pruriginous rash. **.
